# One-Year Infection Eradication and Clinical Outcomes After Completed Two-Stage Revision for Hip and Knee Periprosthetic Joint Infection: A Retrospective Cohort Study

**DOI:** 10.7759/cureus.118884

**Published:** 2026-10-31

**Authors:** Muhindra Rao, Mohd Aizat Azfar Bin Soldin, Maatharasi Balan, Muhammad Azhar Abdullah

**Affiliations:** 1 Orthopaedics, Hospital Sultanah Bahiyah, Alor Setar, MYS; 2 Orthopaedics and Traumatology, Ministry of Health of Malaysia, Selangor, MYS; 3 Orthopaedics, Serdang Hospital, Selangor, MYS

**Keywords:** ambulatory status, clinical outcomes, hip arthroplasty, infection eradication, knee arthroplasty, periprosthetic joint infection, revision arthroplasty, two-stage revision

## Abstract

Background

Two-stage revision is an established treatment for hip and knee periprosthetic joint infection (PJI). Infection eradication is the primary goal, but reconstruction should also provide acceptable pain relief and function. This study evaluated infection eradication and clinical outcomes approximately one year after completed two-stage revision arthroplasty.

Methodology

This retrospective, single-center cohort study included adults who completed two-stage revision for hip or knee PJI between January 2019 and December 2024. The index date was second-stage reimplantation. Successful eradication required no persistent or recurrent PJI, no further surgery for infection, and no suppressive antimicrobial therapy for persistent infection at one year. Secondary outcomes were pain score, ambulatory status, and joint-specific range of motion (ROM). Outcomes were compared by eradication status using the Mann-Whitney U test and the Fisher-Freeman-Halton exact test. These comparisons were exploratory.

Results

Of the 50 patients who underwent second-stage reimplantation, 46 survived and had sufficient follow-up for inclusion in the one-year analysis (24 hip, 22 knee). Successful infection eradication was observed in 41 of these 46 assessed patients (89.1%; 95% confidence interval (CI) = 76.4%-96.4%). The median pain score was 2 (interquartile range (IQR) = 1-2), and 37 (80.4%) patients were independently ambulant at one year. Patients with successful eradication had lower pain scores than those with persistent or recurrent infection (median = 2, IQR = 1-2 vs median = 3, IQR = 2-3; p = 0.012). Ambulatory status also differed between groups (p = 0.002), with independent ambulation in 36 of 41 (87.8%) patients and one of five (20.0%) patients, respectively. No significant differences in hip or knee ROM were detected, although only five patients had persistent or recurrent infection.

Conclusions

Among the 46 assessed survivors after reimplantation, 41 (89.1%) met the one-year infection-eradication criteria. Successful eradication was associated with less pain and greater independent mobility, while joint-specific ROM differences did not reach statistical significance in the small comparison groups. Assessment of pain and ambulation alongside infection status may provide a more clinically meaningful measure of early recovery after septic revision.

## Introduction

Periprosthetic joint infection (PJI) remains one of the most challenging complications of total hip and knee arthroplasty. Patients may require repeated surgery and prolonged antimicrobial therapy. Bone loss, soft-tissue compromise, and impaired mobility can further complicate reconstruction and recovery. The healthcare burden is substantial, and PJI has also been associated with increased mortality [1-3].

Diagnosis and treatment are similarly complex. Contemporary diagnostic frameworks combine clinical findings, inflammatory markers, synovial analysis, microbiology, and intraoperative findings rather than relying on a single investigation [4-8]. When implant exchange is required, the reconstructive strategy must account for the infecting organism, implant stability, host factors, bone stock, and soft-tissue condition.

Two-stage revision remains a widely used treatment strategy for hip and knee PJI [9-12]. The first stage involves removal of the prosthesis and cement, thorough debridement, and placement of an antibiotic-loaded spacer. This is followed by antimicrobial treatment. Definitive reconstruction is undertaken once infection is considered adequately controlled. The pathway therefore exposes the patient to two major operations and a period of impaired function between stages.

Reported infection-control rates after two-stage revision commonly fall between approximately 75% and 90%, although substantial variation exists. Bejon et al. reported an overall success rate of 83%, Romanò et al. reported an average eradication rate of 89.8% after two-stage revision for infected knee arthroplasty, and Bourgonjen et al. reported long-term eradication in 77% of a mixed hip and knee cohort [13-15]. Differences in case mix, follow-up, microbiology, surgical strategy, and definitions of treatment success make direct comparison difficult.

The definition of success is particularly important. Reinfection, further surgery, long-term suppressive antimicrobial therapy, and failure to reach reimplantation are not handled uniformly across published series. An eradication rate therefore has limited meaning unless both the population being studied and the criteria used to define treatment success are clear [13-15].

Control of infection is necessary, but the aim of reconstruction is also to restore a useful and tolerable joint. Previous studies have shown that patients treated for PJI may continue to experience impaired quality of life and functional limitations despite eradication of infection [16-19]. Pain, walking ability, and joint range of motion (ROM) therefore provide useful additional measures of recovery after two-stage revision.

Functional outcome after septic revision is shaped by more than implant survival. Multiple operations, time with a spacer, deconditioning, and soft-tissue compromise may leave a patient with persistent pain or dependence on walking aids despite a retained reconstruction. Published work using quality-of-life and joint-specific scores has demonstrated that this burden can remain after staged revision [16-19]. These observations support the inclusion of simple clinical measures alongside infection status to provide a more comprehensive assessment of treatment outcomes. They also reflect practical questions at follow-up: whether the patient is comfortable, independently mobile, and able to use the reconstructed joint.

The present study evaluated patients who completed two-stage revision arthroplasty for hip or knee PJI at a tertiary orthopedic center. The primary objective was to determine the infection eradication rate at approximately one year following reimplantation. Secondary objectives were to assess pain, ambulatory status, and joint-specific ROM, and to examine whether these outcomes differed according to infection eradication status.

## Materials and methods

Study design and setting

This retrospective, observational, single-center cohort study was conducted at a tertiary orthopedic center in Malaysia. Adult patients who underwent two-stage revision arthroplasty for PJI of the hip or knee between January 1, 2019, and December 31, 2024, were considered for inclusion. Medical and outpatient records were reviewed through December 31, 2025. The date of second-stage reimplantation was defined as the index date for follow-up assessment. All 46 patients included in the analytical cohort underwent clinical assessment at approximately one year after reimplantation, within a window of two to three weeks around the one-year anniversary. This variation reflected routine clinic scheduling constraints, including weekends and public holidays. Infection status, pain, ambulatory status, and joint-specific ROM were assessed at these follow-up visits. Individual follow-up assessment dates were not retained in the analysis dataset; therefore, the exact median follow-up duration and interquartile range (IQR) could not be calculated.

Study population

Patients who underwent first-stage revision for hip or knee PJI between January 1, 2019, and December 31, 2024, were screened for eligibility. Only patients who completed second-stage reimplantation within the study period were eligible for inclusion. PJI was diagnosed according to the 2018 Musculoskeletal Infection Society (MSIS) criteria [7]. Patients were included if they were aged 18 years or older, completed both stages of revision arthroplasty with definitive reimplantation by December 31, 2024, and had documented clinical follow-up at approximately one year after reimplantation.

Patients who did not undergo second-stage reimplantation, died before the one-year assessment, did not have a study-eligible clinical assessment within the approximately one-year follow-up window, or had insufficient clinical records to determine the required outcomes were excluded. Patients treated with debridement, antibiotics, and implant retention; one-stage revision; or definitive resection arthroplasty without reimplantation were not eligible.

Consecutive total population sampling was used. No formal sample size calculation was performed, as all eligible patients within the predefined study period were included.

Data collection

Data were extracted from medical records, operative notes, microbiology reports, relevant investigations, and orthopedic outpatient documentation. Variables included age, sex, affected joint and side, microbiological findings, antimicrobial treatment, operative details, interval between stages, and clinical outcomes at one year. Data that were not documented were recorded as missing and were not imputed.

Ambulatory status at approximately one year was classified from routine clinical documentation into the following three categories: independent ambulation (documented walking without a walking stick), walking aid (documented use of a walking stick or walking frame), and wheelchair use. The need for physical assistance from another person, intermittent or distance-dependent walking-aid use, and mixed mobility patterns were not captured as separate variables in the extracted dataset and therefore could not be assessed reliably. Hip and knee ROM were assessed clinically using a goniometer at the one-year follow-up and were analyzed separately [20].

Surgical and antimicrobial protocol

At first-stage revision, custom-made articulating antibiotic-loaded cement spacers were fashioned intraoperatively. Gentamicin was the most commonly used spacer antibiotic, typically at a dose of 1 g per 40 g of bone cement. Tobramycin or vancomycin was used as an alternative in selected cases. Antibiotic selection was individualized, sometimes following consultation with the infectious diseases team. Patient-level spacer antibiotic formulations and doses were not available for systematic analysis.

Systemic antimicrobial therapy was administered during the interval between first-stage revision and second-stage reimplantation. The usual treatment duration was approximately six weeks, although longer courses were prescribed in selected patients following clinical assessment and discussion with the infectious diseases team. The reported antimicrobial duration represents interstage treatment and excludes any antimicrobial therapy administered after reimplantation.

Second-stage reimplantation was undertaken following clinical assessment of infection control, including wound healing, absence of clinical signs of infection, and improvement or normalization of inflammatory markers (erythrocyte sedimentation rate and C-reactive protein). Intraoperative tissue samples were obtained for microbiological culture at reimplantation when residual infection was clinically suspected; routine reimplantation cultures were not performed in all patients.

Outcome measures

The primary outcome was successful infection eradication at approximately one year following reimplantation. The definition was based on published outcome criteria used following treatment of PJI [15,21,22]. Successful eradication required the absence of documented persistent or recurrent PJI, no further surgical intervention for infection after reimplantation, and no ongoing suppressive antimicrobial therapy for persistent infection at the one-year assessment. Repeat microbiological cultures were recorded where these had been obtained during routine clinical care.

Secondary outcomes were pain score, ambulatory status, and ROM of the affected joint at approximately one year. These measures were selected to assess clinical recovery alongside infection control and have been used in previous studies of two-stage revision [17-19]. Pain was assessed using a routinely documented 0-10 numerical pain scale, where 0 indicates no pain, and 10 indicates the worst possible pain [23]. Hip ROM included flexion, internal rotation, external rotation, and abduction. Knee ROM included extension deficit and flexion.

Statistical analysis

SPSS Statistics for Windows, Version 29 (released 2022; IBM Corp., Armonk, NY, United States) was used for statistical analysis. Continuous variables were assessed for normality using the Shapiro-Wilk test. Normally distributed variables were reported as mean ± standard deviation, while non-normally distributed variables were presented as median and IQR. Categorical variables were reported as frequencies and percentages.

The one-year infection eradication rate was calculated as the proportion of eligible patients meeting the criteria for successful eradication and was reported with an exact 95% binomial confidence interval (CI) using the Clopper-Pearson method.

Pain scores and ROM were compared between patients with successful eradication and those with persistent or recurrent infection using the Mann-Whitney U test. Ambulatory status comprised three categories with small cell counts and was compared using the Fisher-Freeman-Halton exact test. To quantify the magnitude and precision of the observed associations, pain was assessed using a probability-of-superiority effect estimate with a bootstrap 95% CI. Independent ambulation was assessed using the absolute difference in proportions with a Newcombe 95% CI, given the small comparison groups. All statistical tests were two-tailed, with p-values <0.05 considered statistically significant. Comparisons between eradication groups were considered exploratory and interpreted in relation to the available sample size.

Ethical approval

Ethical approval was obtained from the Medical Research and Ethics Committee (MREC), Ministry of Health Malaysia (NMRR ID: NMRR-26-03367-7ML). The requirement for individual informed consent was waived under the approved retrospective study protocol. The study involved retrospective review of existing medical records, with non-identifying patient information handled confidentially in accordance with MREC requirements.

## Results

Cohort characteristics

During the study period, 54 patients underwent first-stage revision for hip or knee PJI. Four patients did not proceed to second-stage reimplantation, leaving 50 patients who completed both stages. Of these, one patient died before the one-year assessment, and three had insufficient follow-up to provide an eligible one-year assessment. The final analytical cohort therefore comprised 46 patients (24 hip and 22 knee) who completed two-stage revision and underwent clinical assessment within approximately two to three weeks of the one-year anniversary following reimplantation (Figure 1).

**Figure 1:**
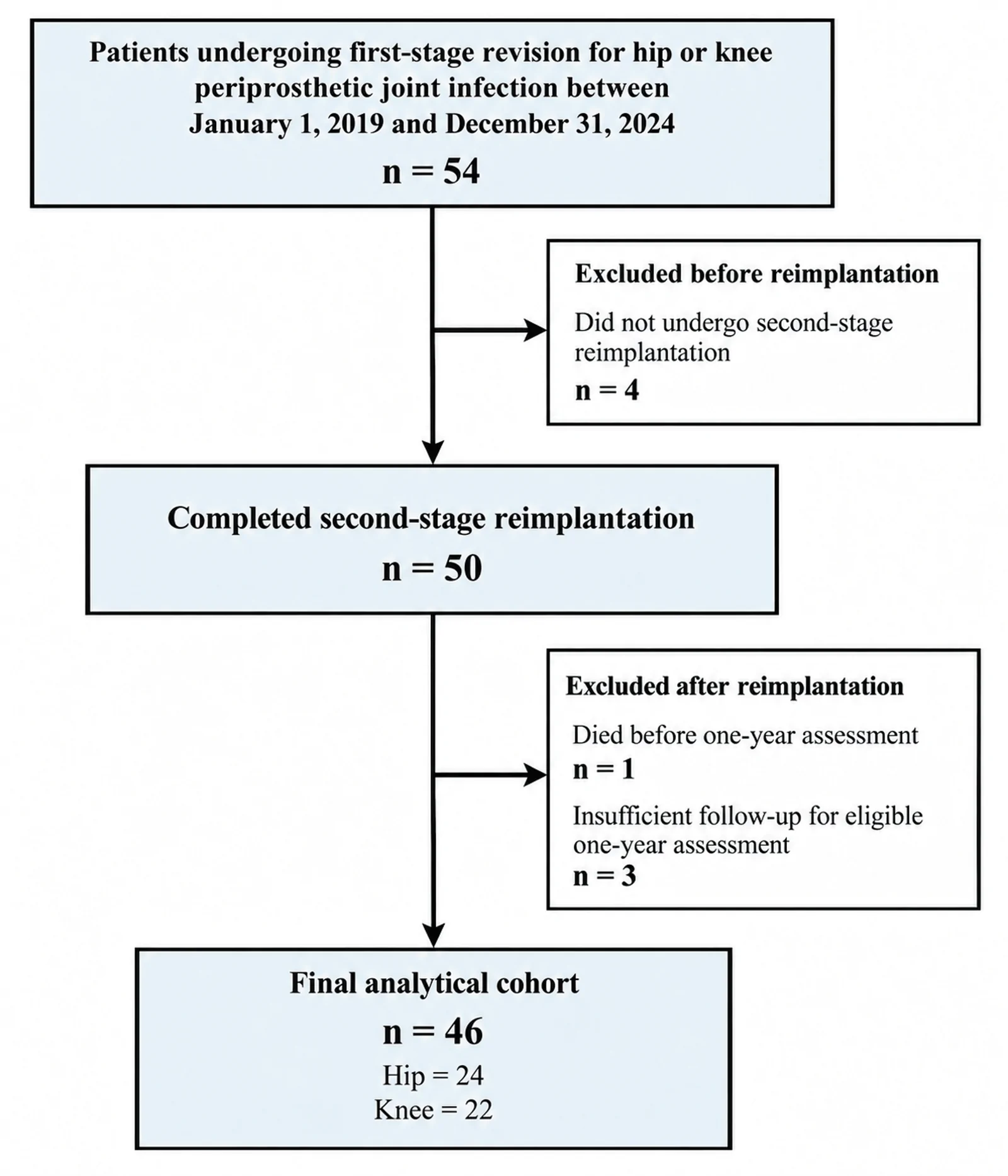
Flow diagram of patient selection. Flow diagram showing selection of the study cohort. Of the 54 patients who underwent first-stage revision for hip or knee periprosthetic joint infection between January 1, 2019, and December 31, 2024, 46 met the inclusion criteria and comprised the final analytical cohort.

The mean age was 63.4 ± 13.0 years, with a mean body mass index (BMI) of 27.2 ± 4.4 kg/m². In total, 25 (54.3%) patients were female. Hip PJI accounted for 24 (52.2%) cases and knee PJI for 22 (47.8%). Baseline characteristics are shown in Table 1.

**Table 1:** Baseline demographic and clinical characteristics (N = 46). BMI: body mass index; SD: standard deviation

Characteristic	Overall cohort
Age, years, mean ± SD	63.4 ± 13.0
BMI, kg/m², mean ± SD	27.2 ± 4.4
Sex, n (%)	
Female	25 (54.3)
Male	21 (45.7)
Affected joint, n (%)	
Hip	24 (52.2)
Knee	22 (47.8)
Laterality, n (%)	
Left	23 (50.0)
Right	23 (50.0)

Microbiology

An organism was isolated in 44 of 46 (95.7%) patients, while two (4.3%) infections were culture-negative. *Staphylococcus aureus* was identified in 16 (34.8%) patients, including three with methicillin-resistant *S. aureus* (MRSA). Coagulase-negative staphylococci were isolated in 14 (30.4%) patients. *Escherichia coli* was identified in 10 (21.7%) patients, of whom five had extended-spectrum beta-lactamase (ESBL)-producing isolates. Three infections were polymicrobial, with methicillin-sensitive *S. aureus* and *E. coli* isolated in the same patient. *Enterococcus faecalis* was identified in three (6.5%) patients, while *Streptococcus agalactiae* and *Pseudomonas aeruginosa* were each identified in two (4.3%) patients. The median duration of antimicrobial therapy was 6.0 weeks (IQR = 6.0-7.0), and the median interval between first-stage revision and reimplantation was 27.5 weeks (IQR = 7.3-51.1) (Table 2). Systemic antimicrobial regimens varied across the cohort. Intravenous antibiotics were documented in all 46 patients; 28 (60.9%) patients had intravenous agents alone recorded, while 18 (39.1%) had both intravenous and oral agents recorded. Intravenous agents included cloxacillin, vancomycin, ampicillin, penicillin, cefazolin, cefuroxime, ciprofloxacin, meropenem, imipenem, and beta-lactam/beta-lactamase inhibitor combinations. Recorded oral agents included ciprofloxacin, rifampicin, clindamycin, cephalexin, linezolid, tedizolid, and trimethoprim-sulfamethoxazole. The available treatment entries did not consistently establish whether intravenous and oral agents were administered sequentially or concurrently.

**Table 2:** Microbiological and treatment characteristics (N = 46). Organism categories are not mutually exclusive because three patients had polymicrobial infection. ESBL: extended-spectrum beta-lactamase; IQR: interquartile range; MRSA: methicillin-resistant *Staphylococcus aureus*; PJI: periprosthetic joint infection

Characteristic	Overall cohort
Culture-positive PJI, n (%)	44 (95.7)
*Staphylococcus aureus*, n (%)	16 (34.8)
MRSA, n/total *S. aureus* (%)	3/16 (18.8)
Coagulase-negative staphylococci, n (%)	14 (30.4)
*Escherichia coli*, n (%)	10 (21.7)
ESBL-producing *E. coli*, n/total *E. coli* (%)	5/10 (50.0)
*Enterococcus faecalis*, n (%)	3 (6.5)
*Streptococcus agalactiae*, n (%)	2 (4.3)
*Pseudomonas aeruginosa*, n (%)	2 (4.3)
Polymicrobial infection (*S. aureus* + *E. coli*), n (%)	3 (6.5)
Culture-negative PJI, n (%)	2 (4.3)
Antimicrobial duration, weeks, median (IQR)	6.0 (6.0–7.0)
Interval to reimplantation, weeks, median (IQR)	27.5 (7.3–51.1)

Infection eradication and clinical outcomes at one year

Successful infection eradication at approximately one year was achieved in 41 of 46 patients (89.1%; 95% CI = 76.4%-96.4%). Five (10.9%) patients had persistent or recurrent infection. Three (6.5%) patients required further surgery for infection after reimplantation, while four (8.7%) were receiving suppressive antimicrobial therapy at the one-year assessment. Two patients underwent further surgery for infection and were also receiving suppressive antimicrobial therapy. The median pain score was 2 (IQR = 1-2). Overall, 37 (80.4%) patients were independently ambulant, six (13.0%) required a walking aid, and three (6.5%) used a wheelchair. Among the 24 patients with hip PJI, median flexion was 110° (IQR = 90°-110°), internal rotation was 30° (IQR = 25°-30°), external rotation was 40° (IQR = 40°-40°), and abduction was 60° (IQR 60°-60°). Among the 22 patients with knee PJI, the median extension deficit was 0° (IQR = 0°-0°), and the median flexion was 100° (IQR = 90°-100°) (Table 3).

**Table 3:** Infection eradication and clinical outcomes at one year. Further surgery and suppressive antimicrobial therapy were not mutually exclusive. CI: confidence interval; IQR: interquartile range; ROM: range of motion

Outcome	Overall cohort
Successful infection eradication, n/N (%)	41/46 (89.1)
95% CI for infection eradication	76.4%–96.4%
Persistent/recurrent infection, n (%)	5 (10.9)
Further surgery for infection, n (%)	3 (6.5)
Suppressive antimicrobial therapy, n (%)	4 (8.7)
Pain score (0–10), median (IQR)	2 (1–2)
Ambulatory status, n (%)	
Independent	37 (80.4)
Walking aid	6 (13.0)
Wheelchair	3 (6.5)
Hip ROM (n = 24), degrees, median (IQR)	
Flexion	110 (90–110)
Internal rotation	30 (25–30)
External rotation	40 (40–40)
Abduction	60 (60–60)
Knee ROM (n = 22), degrees, median (IQR)	
Extension deficit	0 (0–0)
Flexion	100 (90–100)

Pain scores and ambulatory status were available for all 46 patients included in the analysis (41 with successful infection eradication and five with unsuccessful treatment outcomes). All four hip ROM measurements (flexion, internal rotation, external rotation, and abduction) were recorded for the 24 hip patients (21 with successful eradication and three with unsuccessful treatment outcomes). Both knee ROM measurements (extension deficit and flexion) were recorded for all 22 knee patients (20 with successful eradication and two with unsuccessful treatment outcomes). No missing observations were identified for these outcomes in the extracted analysis dataset.

In joint-specific descriptive analyses, successful infection eradication was observed in 21 of 24 (87.5%) hip patients and 20 of 22 (90.9%) knee patients. The median pain score was 2 in both groups, with ranges of 1-3 for hip patients and 1-4 for knee patients. Independent ambulation was recorded in 19 (79.2%) hip patients and 18 (81.8%) knee patients. Walking aids were required by three patients in each group (12.5% and 13.6%, respectively), while two (8.3%) hip patients and one (4.5%) knee patient used a wheelchair. These joint-specific results were descriptive, and no formal hip-versus-knee comparisons were undertaken.

Clinical outcomes according to infection eradication

Patients who achieved infection eradication had lower pain scores at one year. The median pain score was 2 (IQR = 1-2) in the eradicated group compared with 3 (IQR = 2-3) in patients with persistent or recurrent infection (p = 0.012). Ambulatory status also differed between groups (p = 0.002) (Figure 2).

**Figure 2:**
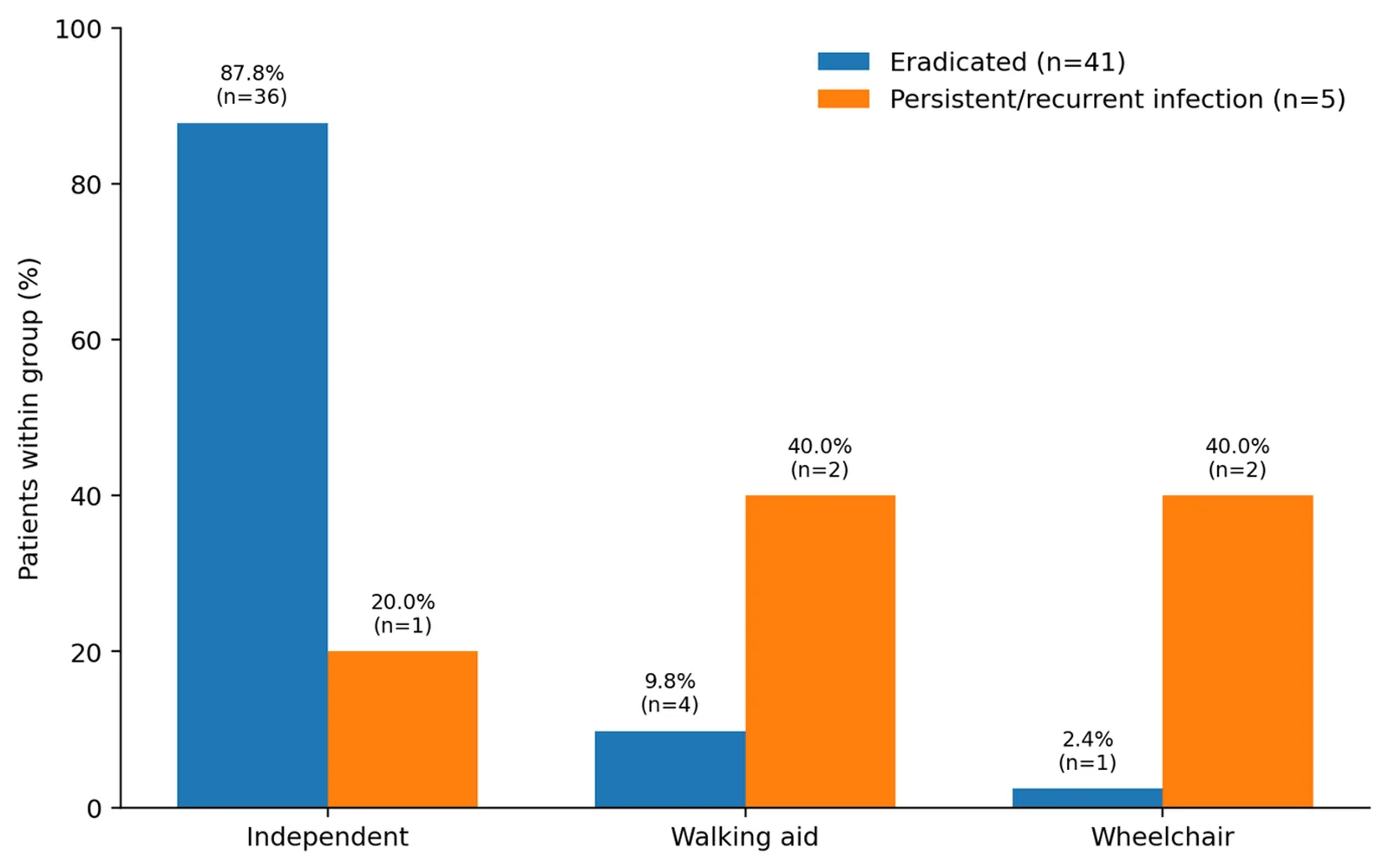
Ambulatory status at approximately one year according to infection eradication status. Bars show the percentage within each eradication group, with patient counts above each bar. Ambulatory status differed between groups on Fisher-Freeman-Halton exact testing (p = 0.002).

Overall, 36 of 41 (87.8%) patients in the eradicated group were independently ambulant, compared with one of five (20.0%) patients with persistent or recurrent infection. In the latter group, two (40.0%) patients required a walking aid and two (40.0%) used a wheelchair. The probability-of-superiority estimate for higher pain scores in the unsuccessful treatment group was 0.82 (bootstrap 95% CI = 0.63-0.96, with ties weighted as one-half). The absolute difference in independent ambulation between the eradicated and unsuccessful treatment groups was 67.8 percentage points (Newcombe 95% CI = 23.3-85.6 percentage points). No statistically significant differences were identified in hip or knee ROM. Median hip flexion was 110° (IQR = 90°-110°) following successful eradication and 110° (IQR = 95°-110°) in patients with persistent or recurrent infection (p = 0.853). Similarly, median knee flexion was 100° (IQR = 90°-100°) and 95° (IQR = 92.5°-97.5°), respectively (p = 1.000). The remaining joint-specific ROM comparisons were also not statistically significant (Table 4).

**Table 4:** Clinical outcomes according to infection eradication status. Pain and ROM were compared using the Mann-Whitney U test. Ambulatory status was compared using the Fisher-Freeman-Halton exact test, for which no U statistic is reported. Hip ROM comparisons included 21 patients with successful eradication and three with persistent or recurrent infection; knee ROM comparisons included 20 and two patients, respectively. IQR: interquartile range; ROM: range of motion; U: Mann-Whitney U statistic

Outcome	Eradicated (n = 41)	Persistent/recurrent infection (n = 5)	U statistic	P-value
Pain score (0–10), median (IQR)	2 (1–2)	3 (2–3)	37	0.012
Ambulatory status, n (%)				
Independent	36 (87.8)	1 (20.0)	—	0.002
Walking aid	4 (9.8)	2 (40.0)		
Wheelchair	1 (2.4)	2 (40.0)		
Hip ROM, degrees, median (IQR)				
Flexion	110 (90–110)	110 (95–110)	34	0.853
Internal rotation	30 (25–30)	30 (30–30)	18	0.211
External rotation	40 (40–40)	40 (40–40)	33	0.902
Abduction	60 (60–60)	60 (60–60)	28.5	0.649
Knee ROM, degrees, median (IQR)				
Extension deficit	0 (0–1.25)	0 (0–0)	25	0.483
Flexion	100 (90–100)	95 (92.5–97.5)	20	1

## Discussion

Among the 46 assessed patients following completed two-stage revision for hip and knee PJI, 41 (89.1%) met the infection-eradication criteria at approximately one year after reimplantation. This observed proportion lies within previously reported ranges; however, differences in patient eligibility, denominators, mortality, follow-up duration, and definitions of treatment success preclude direct comparisons of treatment effectiveness. Bejon et al. reported an 83% success rate, while Bourgonjen et al. reported eradication in 77% of patients at longer follow-up. In a meta-analysis of hip and knee PJI, Goud et al. reported pooled reinfection rates of 8.4% following two-stage hip revision and 16.2% following two-stage knee revision [12-15]. More recent multicenter data have also demonstrated infection eradication rates approaching 90% after two-stage revision of the hip [24].

Comparison between studies requires some care because success after PJI treatment is not defined uniformly. The Delphi consensus, the MSIS outcome criteria, and pragmatic clinical definitions do not classify every patient in the same way [21,22]. Merz et al. further illustrated the importance of outcome definitions: in their two-stage revision subgroup, one-year infection-free survival was 77.6% using Delphi-based criteria and 91.1% using an expanded definition permitting selected subsequent procedures [25]. Our definition focused on the outcomes most relevant after definitive reconstruction: absence of persistent or recurrent infection, no further surgery for infection, and no requirement for suppressive antimicrobial therapy. These criteria are consistent with previously published approaches to reporting successful PJI treatment [15,21,22].

The cohort in this study consisted of patients who completed both stages and underwent definitive reimplantation. This distinction is clinically useful. The study was not designed to estimate attrition across the entire two-stage pathway; rather, it addresses the question faced after reconstruction has been completed: what is the likelihood of maintaining infection control over the subsequent year, and what functional state has the patient reached? Published studies that include patients who never undergo reimplantation naturally report lower overall pathway success [26-28]. The observed eradication proportion therefore provides clinically relevant information for counselling patients who have reached definitive reconstruction. However, it must be interpreted as an early post-reimplantation outcome among assessed survivors, rather than an estimate of treatment success for all patients entering or completing the two-stage revision pathway.

The functional findings provide additional descriptive information regarding outcomes after two-stage revision. Patients meeting the study’s infection-eradication criteria had lower pain scores and were more frequently independently ambulant at approximately one year. Independent ambulation was observed in 36 of 41 (87.8%) patients with successful eradication, compared with one of five (20.0%) patients with unsuccessful treatment outcomes. However, these findings represent unadjusted associations rather than evidence of a causal relationship between infection eradication and functional recovery. Baseline mobility, frailty, comorbidities, previous surgical procedures, and reconstructive complexity may have influenced both treatment outcomes and postoperative function. These potential confounding factors were not accounted for in the comparative analysis. Accordingly, the observed differences should be interpreted as exploratory associations, while pain and ambulatory status remain useful complementary measures of clinical recovery. Persistent pain or impaired mobility after arthroplasty should not automatically be attributed to infection alone, as noninfectious neurological conditions may also contribute to postoperative symptoms [29].

This relationship between infection control and patient function has also been observed elsewhere. Poulsen et al. found that patients who developed reinfection after completed two-stage hip revision had poorer health-related quality of life than those who remained infection-free [17]. Studies of staged revision have similarly shown persistent physical health deficits after PJI treatment and meaningful improvement in pain and joint function after successful reconstruction [16-19]. Our findings extend this observation using simple clinical measures that are readily available during routine arthroplasty follow-up.

ROM provided a different perspective on postoperative recovery. Although numerical differences were observed in some joint-specific ROM measurements, none reached statistical significance (Table 4). This finding is clinically plausible, as joint movement following definitive reconstruction is influenced by several factors beyond infection control, including pre-existing stiffness, previous surgical procedures, bone and soft-tissue reconstruction, implant choice, and rehabilitation. Nevertheless, the small number of patients with unsuccessful treatment outcomes (three hips and two knees) limits the precision of these comparisons, and the absence of statistical significance should not be interpreted as evidence of equivalent ROM between groups. Winkler et al. similarly assessed ROM alongside pain, joint function, and quality of life, illustrating that these measures represent related but distinct aspects of recovery [19]. In the present cohort, pain scores (p = 0.012) and ambulatory status (p = 0.002) differed significantly according to infection-eradication status, whereas joint-specific ROM measurements did not. This clinically interesting observation suggests that pain and walking ability may provide complementary insights into functional recovery following two-stage revision, although further studies are needed to establish their relative discriminatory value.

The microbiological profile also reflects the complexity encountered in revision PJI practice. *S. aureus* and coagulase-negative staphylococci accounted for a large proportion of infections, while *E. coli* was identified in more than one-fifth of the cohort. Three of the 16 *S. aureus* infections were MRSA, and half of the *E. coli* isolates were ESBL-producing. Resistant organisms are particularly relevant in staged revision because they narrow antimicrobial options and have been associated with poorer infection control outcomes in some series. In a multicenter study of two-stage hip revision, antibiotic-resistant organisms and polymicrobial infection were associated with reinfection [24]. Despite the presence of resistant organisms in our cohort, the overall one-year eradication rate remained high. The present study was not powered to determine organism-specific failure risk, and these microbiological findings are therefore best interpreted as a description of the treatment burden rather than evidence that resistance did or did not influence outcome.

The median interval between first-stage revision and reimplantation was 27.5 weeks (IQR = 7.3-51.1), representing a relatively prolonged treatment period with considerable variability. In clinical practice, the timing of reimplantation may be influenced by several factors, including infection control, medical optimization, implant procurement, operating theater availability, and delays in securing funding for revision implants. These clinical and logistical considerations may contribute to extended intervals between stages, particularly in resource-constrained healthcare settings, including our institution. However, as the reasons for delayed reimplantation were not systematically evaluated in this study, their individual contributions could not be determined.

This study has several limitations. Its retrospective, single-center design and relatively short follow-up may limit the generalizability of the findings and underestimate late infection recurrence. The analysis was restricted to patients who completed reimplantation and survived to the one-year assessment, introducing potential selection and survivor bias. Only five patients had persistent or recurrent infection, limiting subgroup comparisons and preventing meaningful multivariable analysis. The comparisons were exploratory and unadjusted. Baseline mobility, frailty, comorbidities, previous surgical procedures, and reconstructive complexity were not controlled for in the analysis and may have contributed to the observed associations between infection eradication status and functional outcomes. Consequently, causal conclusions cannot be drawn from these findings. Detailed patient-level spacer antibiotic formulations and doses were not systematically available. Furthermore, reimplantation cultures were obtained selectively rather than routinely, and the number of patients cultured and the number of positive cultures at reimplantation could not be reliably determined from the available dataset. Consequently, microbiological findings at reimplantation could not be analyzed. Ambulatory status was derived from routine clinical documentation rather than a standardized functional assessment, and the absence of information on human assistance or variable walking-aid use may have introduced classification uncertainty. Despite these limitations, the study provides one-year clinical outcomes following completed two-stage revision, using routinely documented measures of pain, ambulation and ROM. These findings offer a practical assessment of recovery alongside infection control.

Taken together, the findings support a broader view of outcome after two-stage revision. Among the 46 assessed patients, almost nine in ten met the one-year infection-eradication criteria, and those meeting these criteria generally experienced less pain and were more independently mobile. The joint-specific ROM comparisons did not reach statistical significance, although interpretation was limited by the small unsuccessful treatment subgroups. For clinical practice, this suggests that follow-up after septic revision should consider infection status together with pain and walking ability when judging the early success of reconstruction.

## Conclusions

Of the 50 patients who underwent second-stage reimplantation, 46 were included in the one-year analysis after excluding one patient who died and three with insufficient follow-up. Among these 46 assessed patients, 41 (89.1%) met the study’s infection-eradication criteria. Patients with successful eradication had lower pain scores and were substantially more likely to walk independently. Although numerical differences in hip and knee ROM were observed, these did not reach statistical significance in the small comparison groups. These findings support assessment of recovery after septic revision beyond infection control alone. Pain and ambulatory status appear to provide clinically useful information about early post-reimplantation outcome and can be readily incorporated into routine arthroplasty follow-up. Longer follow-up is required to determine the durability of infection control and to identify late recurrence.
